# Silicon-Mediated Resistance in a Susceptible Rice Variety to the Rice Leaf Folder, *Cnaphalocrocis medinalis* Guenée (Lepidoptera: Pyralidae)

**DOI:** 10.1371/journal.pone.0120557

**Published:** 2015-04-02

**Authors:** Yongqiang Han, Wenbin Lei, Lizhang Wen, Maolin Hou

**Affiliations:** 1 College of Plant Protection, Hunan Agricultural University, Changsha, Hunan Province, China; 2 State Key Laboratory for Biology of Plant Diseases and Insect Pests, Institute of Plant Protection, Chinese Academy of Agricultural Sciences, Haidian, Beijing, China; South China Agricultural University, CHINA

## Abstract

The rice leaf folder, *Cnaphalocrocis medinalis* (Guenée), is one of the most destructive rice pests in Asian countries. Rice varieties resistant to the rice leaf folder are generally characterized by high silicon content. In this study, silicon amendment, at 0.16 and 0.32 g Si/kg soil, enhanced resistance of a susceptible rice variety to the rice leaf folder. Silicon addition to rice plants at both the low and high rates significantly extended larval development and reduced larval survival rate and pupation rate in the rice leaf folder. When applied at the high rate, silicon amendment reduced third-instars’ weight gain and pupal weight. Altogether, intrinsic rate of increase, finite rate of increase and net reproduction rate of the rice leaf folder population were all reduced at both the low and high silicon addition rates. Although the third instars consumed more in silicon-amended treatments, C:N ratio in rice leaves was significantly increased and food conversion efficiencies were reduced due to increased silicon concentration in rice leaves. Our results indicate that reduced food quality and food conversion efficiencies resulted from silicon addition account for the enhanced resistance in the susceptible rice variety to the rice leaf folder.

## Introduction

The rice leaf folder, *Cnaphalocrocis medinalis* (Guenée) (Lepidoptera: Pyralidae), one of the most destructive insect pests on rice, is distributed widely in rice-growing regions of Asia, Madagascar, northeastern Australia and Oceania [1]. This insect damages rice crops during its larval stage. The larva folds a leaf blade longitudinally with silk strands and feeds on mesophyll tissue inside the folded leaf, thereby creating longitudinal white and transparent streaks on the blade, disturbing photosynthesis and growth and ultimately reducing rice yield [2–4]. Serious outbreaks of the rice leaf folder have been reported in many Asian countries [2]. Since 2003, occurrence of the rice leaf folder has been seriously increasing in China, resulting in infestation of more than 20 million hectare and rice yield loss of up to 760 million kg per year [5]. Currently, the rice leaf folder populations are principally managed with chemical insecticides. However, in addition to causing the so-called ‘3R’ problems, these chemicals have not achieved the desired control [6], largely due to the insect’s shelter inside a folded leaf blade and its migratory behavior, the latter necessitating precise timing and repeated insecticide applications. Therefore, it is imperative to develop an alternative approach to control this pest. Cultivar resistance and crop management are currently the dominant tactics being developed.

One crop management tactic that can benefit the control of insect pests is the amendment of a silicon (Si) fertilizer [7–18]. Although Si is generally not considered an essential element in higher plants, Si-accumulating gramineous plants, if deprived of Si, may be afflicted with a range of abnormalities in growth, development, viability and reproduction [7,18,19] and be more susceptible to abiotic and biotic stresses [20,21]. There is increasing evidence showing positive association between high plant Si content and variety resistance to insect herbivory in monocots and dicots [21–35]. In most cases, plant resistance to herbivores can also be achieved through Si amendment to the host plants [7–18].

Si amendment can influence insect herbivores in several ways. Si is deposited primarily in the epidermal layers of the leaf sheath and blade to form a cuticle-silica double layer [8] or in vascular and other tissues associated with protection, storage, support and strengthening [36], thus creating physical barriers to herbivores. For the Asiatic rice borer, *Chilo suppressalis* (Walker), Si addition to rice plants significantly deterred larval boring behavior [16], prolonged larval development and decreased weight gain and stem damage by the insect [9]. Si addition can also impede an insect herbivore’s food consumption efficiency and thus retard the insect’s performance. Larvae of *Spodoptera exempta* (Walker) and *Schistocerca gregaria* (Forskål), when fed on Si-amended plants of three grass species, showed reduced efficiency of conversion of ingested food and reduced relative growth rate [13]. Similar results were reported for the sugarcane borer, *Diatraea saccharalis* (Fabricius), where Si amendment led to low relative growth rates and reduced boring success [10]. Further, Si amendment may result in differential regulation of genes and alter metabolism in plants, with subsequent effects on pathogens and herbivores, as evidenced by Brunings et al. [37], who reported that Si amendment differentially regulated 221 genes in rice and concluded that Si affected rice response to infection by a pathogenic fungus at a transcriptional level.

Rice is typically a Si-accumulating plant species. It is estimated that a rice crop producing 1000 kg of yield will remove 130 kg Si from the soil, twice as much as the absorption of nitrogen, phosphorus and potassium nutrients combined [8]. For the rice leaf folder, resistant rice varieties have been reported to show either a high density of silica cells or high Si content in the leaf blade [25–26,29–33]. Ye ta al. [38] showed that rice leaf folder larvae fed on Si-treated wild type rice plants gained less mass and proved that the observed Si-mediated resistance occurred in a jasmonate-dependent manner. However, detailed information of the effects of Si addition on rice plant resistance to the rice leaf folder is still scarce.

In this study, we aimed to determine 1) the effects of Si addition on resistance of a rice variety susceptible to the rice leaf folder through detailed evaluation of development, reproduction and population parameters and 2) the mechanisms for Si-mediated resistance through measurement of feeding and food conversion parameters. Such information may help provide evidence of Si-mediated resistance to the rice leaf folder and improve management of the pest in rice production.

## Materials and Methods

### Plants and Si Treatments

Seeds of a susceptible variety (Taichung Native 1, TN1) [30] were incubated in 50°C water for 24 h and germinated for 72 h in a climate chamber at 28°C before seeding. Then seeds were sown in soil without addition of calcium silicate. The soil consisted of a sandy loam, with pH = 5.7, organic C = 2.19%, total N = 1.58 g/kg, available P = 3.22 mg/kg, available Si = 0.21 g/kg and available K = 98.16 mg/kg. The rice seedlings were transplanted to 10 L PVC pots (24.5 cm in diameter and 20.0 cm in height) 25 days after sowing at two 2-seedling hills per pot in a glasshouse at Guilin Experiment Station for Crop Pests (25° 36' 00" N, 110° 41' 24" E), Ministry of Agriculture, China. Each pot contained 4.2 kg dry soil, amended with calcium silicate (soluble Si ≥ 11.7%, Shanxi Fubon Siliconfat Co., Ltd, China) at a low rate of 0.16 or a high rate of 0.32 g Si/kg soil or left untreated (the control). All the pots were treated with urea (N ≥ 46.4%), diammonium phosphate (N = 16.0%; P_2_O_5_ = 44.0%) and potassium chloride (K_2_O ≥ 60.0%) at a rate of 0.37 g/kg soil, 0.25 g/kg soil and 0.35 g/kg soil, respectively. Urea was applied into soil 3 d before transplanting or top dressing at tillering, heading and milk stages at a ratio of 4:3:2:1, diammonium phosphate and calcium silicate were all incorporated into the soil 3 d before transplanting, and potassium chloride was supplied in soil 3 d before transplanting or top dressing at heading stage at a ratio of 2:1 [9]. The pots were arranged randomly in the glasshouse. Watering was administered as necessary and water level in the pots was always below the upper edge. Pesticides were not used throughout the experiment.

### Insects

Rice leaf folder adults were collected in late June, 2013, from paddy fields at the experiment station. The adults were confined to caged rice plants in the field for oviposition. Eggs together with segments of leaf blades were collected daily from the plants and placed on moistened filter paper in a Petri dish (15 cm in diameter and 2 cm in height). Newly hatched first instars (< 24 h) and newly molted third instars (< 24 h) were used in the experiments.

### Development and Survival

A leaf segment method was employed to determine the effects of Si amendment on larval development and survival of the rice leaf folder. A segment (12 cm long) of the reciprocal fourth leaves was cut from a 40-DAT (day after transplanting) rice plant and lined on a moistened filter paper in the Petri dish at 5 leaf segments per dish, with the segment ends wrapped with moistened wool. Twenty-five newly hatched first instars were transferred onto the leaf segments in a Petri dish with a pointed fine camel hair brush. The insects were left to develop in a climate chamber at 28 ± 1°C, 70 ± 5% relative humidity (RH) and a photoperiod of 16:8 (L:D) h. The dishes were observed twice daily (at 0800 and 1800 hours) to record larval stage. Numbers of surviving larvae were counted and dead individuals were removed daily. The leaf segments were replaced every 48 h until pupation. Eight-day-old larvae and newly formed pupae were weighed individually using an electronic balance (Model AL 240-IC, Mettler, Shanghai) to the nearest of ± 0.1 mg. Larval duration, survival rate and pupation rate were calculated from the recorded data. Pupae resulting from a treatment were dated, sexed under a microscope and transferred to a Petri dish. The pupae were observed daily for emergence, and numbers of emerged adults were recorded. Pupal duration, emergence rate and sex ratio were calculated.

### Food Consumption Efficiency of Third Instars

A segment (7 cm long) of the reciprocal fourth leaves was cut from a 40-DAT rice plant. After being weighed, the leaf segment was lined on a moistened filter paper in a Petri dish (9 cm in diameter and 2 cm in height) at three segments per dish, with the segment ends wrapped with moistened wool. A newly molted third instar (< 24 h), starved for 6 h and weighed, was transferred onto the leaf segments in the Petri dish. The insect was left to feed inside the dish in the climate chamber for 72 h. Then it was starved for another 6 h to allow all the feces to be evacuated. After being weighed again, the insect was dried at 80°C to a constant weight and then weighed. The remaining leaf segments were cleared of feces and then dried at 80°C for 48 h and weighed. Feces were collected, also dried and weighed.

Food consumption parameters, expressed on the basis of dry weight [13,39], were calculated for each treatment. For calculation of dry weight of leaves before feeding tests, 10 segments (7 cm long) of the reciprocal fourth leaves, each cut from a 40-DAT rice plant in a treatment and weighed individually, were dried at 80°C to a constant weight and then weighed individually for dry weight. Water contents of leaf segments and rice leaf folder larvae were calculated as the differences between fresh and dry weight and used to convert the initial fresh mass before feeding test to dry mass. The parameters included food consumed (*FC*, mg/d) = *E*/*T*, relative consumption rate (*RCR*) = *E*/(*T* × *A*), relative growth rate (*RGR*) = *P*/(*T* × *A*), approximate digestibility (*AD*, %) = 100 × (*E*–*F*)/*E*, efficiency of conversion of ingested food (*ECI*, %) = 100 × (*P*/*E*) and efficiency of conversion of digested food (*ECD*, %) = 100 × *P*/(*E*–*F*), where: *A* = average of initial and final dry weights of the larvae in a feeding period (mg), *E* = dry weight of food ingested (mg), *F* = dry weight of feces produced (mg), *P* = gain in larval dry weight in a feeding period (mg), *T* = duration of feeding period (d).

### Fecundity and Viability of F1 Eggs

Three pairs of 1-d-old male and female adults obtained from a treatment were confined in an oviposition cage (27 cm in diameter and 85 cm in height) with 40-DAT rice plants of their original treatment and supplied with a ball of cotton wool soaked in honey solution (30% w/v). Numbers of deposited eggs were recorded and leaves with eggs were removed daily until all the adults died.

At peak oviposition, about 200 eggs laid on the same day were collected together with a leaf segment from each treatment and placed on a moistened filter paper in a Petri dish (15 cm in diameter and 2 cm in height). Numbers of hatched larvae were recorded daily until there was no hatching.

### Determination of Si, Soluble Sugar and Nitrogen Contents

For determination of Si content in leaves and stems and soluble sugar and nitrogen contents in rice leaves, the reciprocal fourth leaves (about 100 g) and stems (including culm and leaf sheath, about 200 g) were collected from rice plants not fed by the rice leaf folder at 40 DAT. The samples were flushed with tap water to get rid of soil and were dried at 105°C for 30 min and further dried at 70°C for 48 h. The dried samples were ground using a mortar grinder (ZM200, Retsch GmbH, Germany). The resulting sample powder was used in determination of Si content in leaves and stems by the colorimetric molybdenum blue method [40], and soluble sugar and nitrogen contents in leaves by the anthrone method [41] and the Kjeldahl method [41], respectively. C:N ratio was calculated from the determined soluble sugar and nitrogen contents.

### Data Analysis and Statistics

Data were subjected to one-way analysis of variance (ANOVA), followed by Tukey’s multiple range test (*P* = 0.05) for significant differences between treatments [42]. Percentage data were arcsine square-root transformed, and homogeneity of variance of all data was tested before performing ANOVA [42]. Life table statistics, including the intrinsic rate of increase (*r*_*m*_), finite rate of increase (*λ*), net reproductive rate (*R*_*0*_), and mean generation time (*T*) were calculated for populations at different Si treatments using the computer program [43]. Differences in the life table statistics among Si treatments also were subjected to ANOVA and separated using Tukey’s multiple range tests.

## Results

### Development, Survival and Reproduction

**Fig 1:**
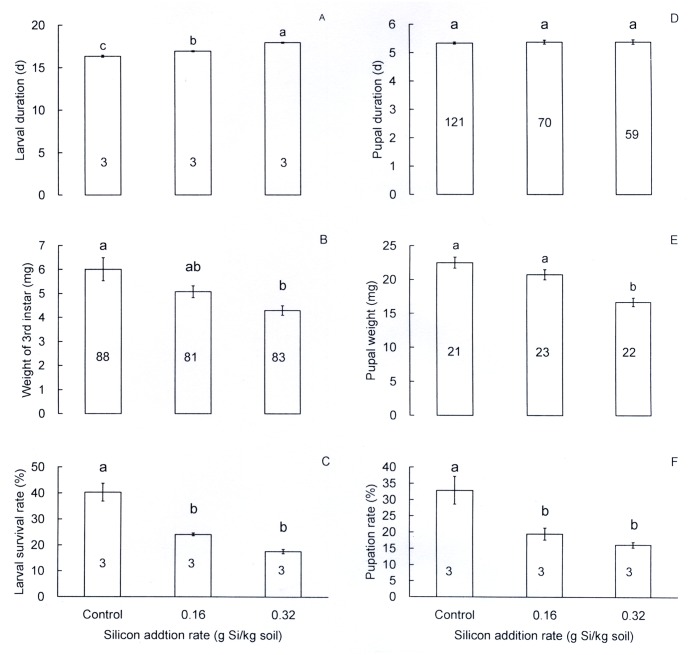
Effects of silicon addition to rice plants (TN1) on growth and development of *Cnaphalocrocis medinalis* larvae and pupae. A: Larval duration, B: weight of third instars, C: larval survival rate, D: pupal duration, E: pupal weight, F: pupation rate. Values are expressed as means ± SE. Bars with different letters are significantly different (Tukey’s multiple range test, *P* = 0.05). Numbers in bars indicate replications. Larval duration, larval survival rate and pupation rate were observed to three 150-first-instar groups in each treatment.

Duration of rice leaf folder larvae differed significantly between Si treatments (*F* = 74.97, df = 2,6, *P* < 0.001; Fig. 1A). Si amendment at the high and low Si addition rates significantly extended larval development by 9.7% and 3.6%, respectively, from that in the control. Third instars at the high Si addition treatment weighed less (by 28.6%) than those in the control (*F* = 6.51, df = 2,249, *P* = 0.002; Fig. 1B). Si amendment at both the high and low rates significantly decreased larval survival rate (*F* = 36.09, df = 2,6, *P* < 0.001; Fig. 1C) by 22.7% and 16.2%, respectively, as compared with the control. Si amendment had no significant effects on pupal duration (*F* = 0.12, df = 2,247, *P* = 0.89; Fig. 1D) while pupal weight was significantly reduced by 25.9% at the high Si addition rate from that in the control (*F* = 16.54, df = 2,63, *P* < 0.001; Fig. 1E). Pupation rate was also significantly reduced by Si addition (*F* = 11.26, df = 2,6, *P* = 0.009; Fig. 1F), by 16.7% at the high and 13.4% at the low Si addition rate in comparison with the control.

**Fig 2:**
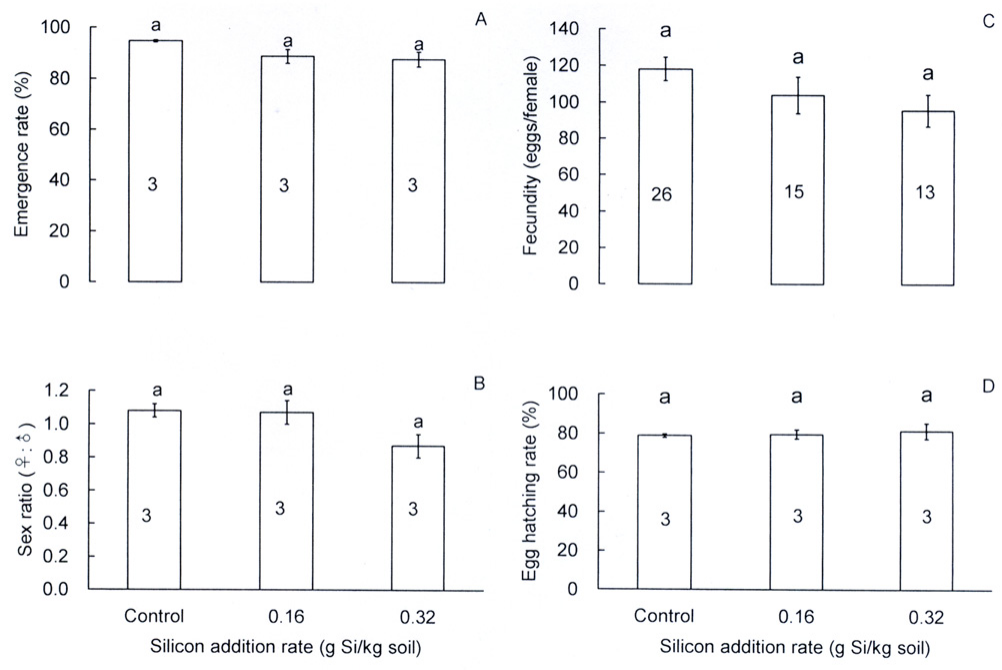
Effects of silicon addition to rice plants (TN1) on adult emergence and reproduction of *Cnaphalocrocis medinalis*. A: Emergence rate, B: sex ratio, C: fecundity, D: egg hatching rate. Values are expressed as means ± SE. Bars with different letters are significantly different (Tukey’s multiple range test, *P* = 0.05). Numbers in bars indicate replications. Emergence rate and sex ratio were based on three 30-pupa groups in each treatment. Egg hatching rate was observed to three 200-egg groups in each treatment.

Si treatment showed no significant influence on emergence rate (*F* = 3.83, df = 2,6, *P* = 0.085; Fig. 2A), sex ratio (*F* = 4.03, df = 2,6, *P* = 0.078; Fig. 2B), fecundity (*F* = 2.11, df = 2,77, *P* = 0.132; Fig. 2C).and viability of F1 eggs (*F* = 0.273, df = 2,6, *P* = 0.77; Fig. 2D).

### Life Table Parameters

With the obtained data, life table parameters of the rice leaf folder fed on different Si treatments were calculated (Table 1). Si treatment exerted significant influence on the intrinsic rate of increase (*F* = 85.295, df = 2,6, *P* < 0.001), the finite rate of increase (*F* = 87.369, df = 2,6, *P* < 0.001) and the net reproduction rate (*F* = 49.858, df = 2,6, *P* < 0.001) of the rice leaf folder. At the high and low Si addition rates, the intrinsic rates of increase were significantly reduced by 41.9% and 28.4%, respectively; the finite rates of increase, by 3.7% and 2.8%, respectively; and the net reproductive rates, by 63.3% and 49.9%, respectively, from those in the control (Table 1). The mean generation time was not different between Si treatments (*F* = 4.518, df = 2,6, *P* = 0.064).

**Table 1:** A life table analysis of population parameters of *Cnaphalocrocis medinalis* for effects of silicon treatment to rice plants.

Population parameters	Silicon treatment (g Si/kg soil)		
	0	0.16	0.32
*r*_*m*_	0.074 ± 0.001 a	0.053 ± 0.002 b	0.043 ± 0.002 c
*λ*	1.08 ± 0.001 a	1.05 ± 0.002 b	1.04 ± 0.002 c
*R*_*0*_	13.19 ± 0.92 a	6.61 ± 0.43 b	4.84 ± 0.37 b
*T*	34.68 ± 0.56 a	35.64 ± 0.35 a	36.48 ± 0.33 a

Data are expressed as mean ± SE. Data in a row followed by different letters are significantly different (Tukey’s multiple range test, *P* = 0.05). The observation was made to three populations in each treatment. *r*_*m*_: intrinsic rate of increase (eggs per female per d), *λ*: finite rate of increase (population growth rate per d), *R*_*0*_: net reproductive rate (eggs per female), *T*: mean generation time (days).

### Food Consumption Efficiency of Third Instars

**Fig 3:**
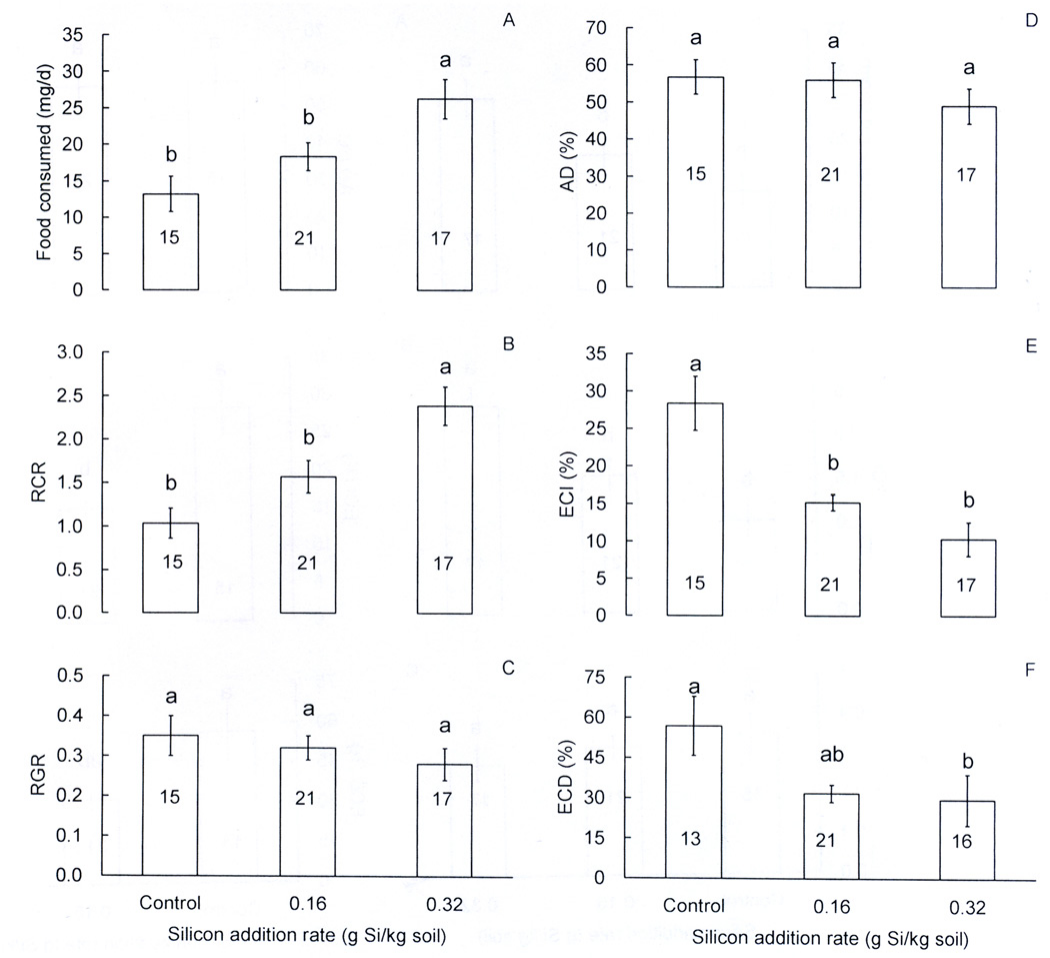
Effects of silicon addition to rice plants (TN1) on food consumption efficiency in third instars of *Cnaphalocrocis medinalis*. A: Food consumed, B: relative consumption rate (*RCR*), C: relative growth rate (*RGR*), D: approximate digestibility (*AD*), E: efficiency of conversion of ingested food (*ECI*), F: efficiency of conversion of digested food (*ECD*). Values are expressed as means ± SE. Bars with different letters are significantly different (Tukey’s multiple range test, *P* = 0.05). Numbers in bars indicate replications.

Si addition at the high rate significantly increased (by 98.5%) the amount of food consumed by rice leaf folder third instars (*F* = 7.31, df = 2,50, *P* = 0.002; Fig. 3A) over the control. The relative consumption rate (*RCR*) at the high Si addition rate was 129.8% higher than in the control (*F* = 10.93, df = 2,50, *P* < 0.001; Fig. 3B). No significant differences were observed for both the relative growth rate (*RGR*) (*F* = 0.774, df = 2,50, *P* = 0.466; Fig. 3C) and approximate digestibility (*AD*) (*F* = 0.91, df = 2,50, *P* = 0.408; Fig. 3D) between the treatments. Si amendment significantly reduced the efficiency of conversion of ingested food (*ECI*) (*F* = 16.91, df = 2,50, *P* < 0.001; Fig. 3E) and efficiency of conversion of digested food (*ECD*) (*F* = 7.07, df = 2,48, *P* = 0.002; Fig. 3F) in the third instars. *ECI* was 18.0% and 13.1% lower at the high and low Si addition rates, respectively, than in the control. For *ECD*, these reductions were 27.4% and 25.1%, respectively.

### Si and Soluble Sugar Contents and C:N Ratio

**Fig 4:**
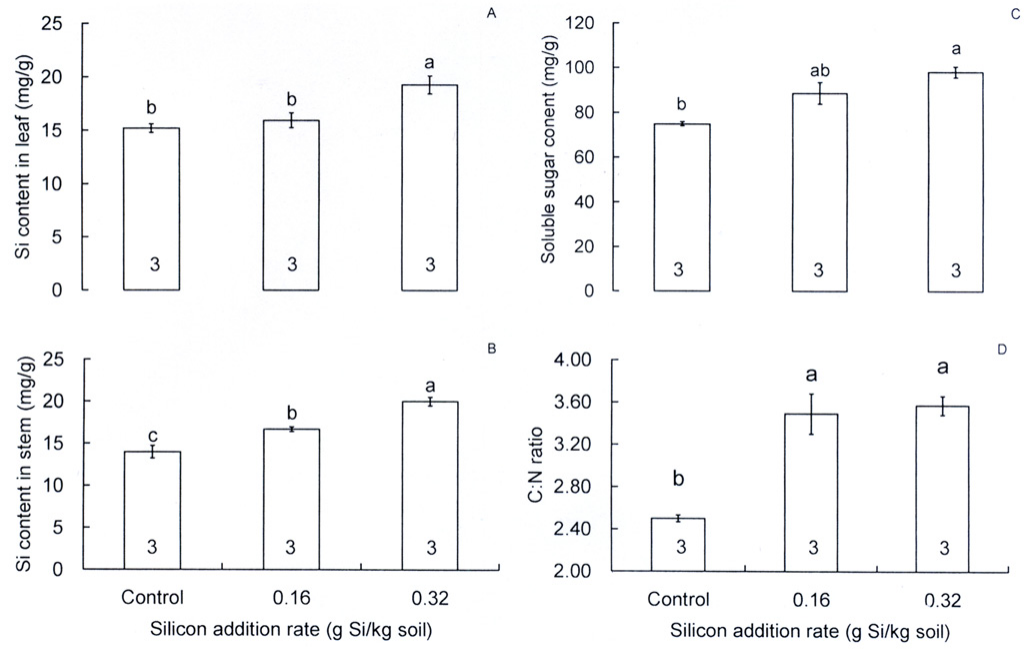
Effects of silicon addition on silicon and soluble sugar content and C:N ratio in the reciprocal fourth leaves and stems. A: Silicon content in rice leaves, B: silicon content in rice stems, C: soluble sugar content in rice leaves, D: C:N ratio in rice leaves. Values are expressed as means ± SE. Bars with different letters are significantly different (Tukey’s multiple range test, *P* = 0.05). Numbers in bars indicate replications.

Si amendment at the high rate significantly increased Si contents (by 26.7%) in rice leaves over that in the control (*F* = 10.19, df = 2,6, *P* = 0.012; Fig. 4A). In rice stems, Si content was increased significantly (*F* = 30.43, df = 2,6, *P* = 0.001; Fig. 4B) by 42.9% and 19.1% at the high and low Si addition rates over that in the control, respectively. Soluble sugar content was enhanced (*F* = 13.255, df = 2,6, *P* = 0.006; Fig. 4C) by 30.9% and 18.3% at the high and low Si addition rates from the control, respectively, while C:N ratio was increased (*F* = 23.703, df = 2,6, *P* = 0.001; Fig. 4D) by 39.6% and 42.8% at the the high and low Si addition rates from the control, respectively.

## Discussion

Rice is one of the typical Si-accumulating plant species, and Si accumulation in rice is an active process [44]. In this study, Si contents in both leaves and stems in the susceptible rice variety TN1 increased in response to Si amendment, which is in conformity with previous reports [9–16,45]. We recorded higher increases in Si content in stems than in the reciprocal fourth leaves, which confirmed a previous report by Dai et al. [46]. Such differences may lead to differential responses from herbivores with varying feeding habits and positions.

The increased Si content from Si amendment conferred resistance to the rice leaf folder, as indicated by the reduced intrinsic rate of increase, finite rate of increase and net reproduction rate of the rice leaf folder population. Enhanced plant defense associated with Si amendment has been recently reported for the rice leaf folder [38], and also for other herbivores, such as the Asiatic rice borer, *C*. *suppressalis* [9,16], the sugarcane borer, *D*. *saccharalis* [10], the African stem borer, *Eldana saccharina* Walker [11,12,15,17], the African armyworm, *S*. *exempta* [13] and the desert locust, *S*. *gregaria* [13].

As reported for crops within the family Poaceae, increased resistance associated with Si addition may result from a cuticle-silica double layer, which is formed in the leaf blade and acts as a mechanical barrier to insect herbivores [8]. Furthermore, rice varieties resistant to the rice leaf folder have closer silica chains, heavy deposition of silica in the intercostal area, high epidermal silica deposition and a single or double row of silica, in contrast to susceptible rice varieties [28]. In several species of gramineous grasses, Si addition increased leaf abrasiveness, which reduced the feeding activity of *S*. *exempta* and *S*. *gregaria* [13]. In the present study, rice leaf folder larvae consumed more rice leaves on Si-treated plants than on control plants, which is in contrast to our results for the Asiatic rice borer, where Si amendment deterred stem-boring behavior [16]. The difference in feeding damage by the two pests may be due to their different feeding habits (boring into stem versus feeding on mesophyll tissue of leaves) and the higher deposition of Si in rice stems than in the reciprocal fourth leaves in response to Si amendment (Fig. 4A, 4B). The increased feeding amount on Si-treated plants versus on control plants indicates that the Si-associated mechanical barrier mechanism was not effective or that Si addition did not improve such mechanism against the rice leaf folder.

Si addition may lead to changes in nutrient contents or digestibility of host plants and influence food conversion efficiencies in herbivores, which may affect herbivores’ performance. In the current study, the high rate Si addition significantly increased food consumed and relative consumption rate (*RCR*), whereas significantly reduced efficiency of conversion of ingested food (*ECI*) and efficiency of conversion of digested food (*ECD*) in third instars of the rice leaf folder. Increased consumption as a result of Si addition has been also reported for *Spodoptera eridania* (Cramer) (Lepidoptera: Noctuidae) [47] and *S*. *gregaria* [13]. Reduced *ECI* is also recorded in the larvae of *S*. *exempta* and *S*. *gregaria* fed on Si-amended grass plants [13]. Massey & Hartley [14] further showed that Si addition reduced both the efficiency with which *S*. *exempta* converted ingested food into body mass and the amount of nitrogen absorbed from the diet. We found a positive response of soluble sugar concentration to Si addition (Fig. 4C), Dallagnol et al. [45] also reported an increased soluble sugar concentration as a result of Si addition. Although soluble sugars are generally phagostimulants to most insects [48], the enhanced sugar concentration may dilute nitrogen concentration and result in a low quality food (increased C:N ratio, Fig. 4D). Therefore, the increased consumption by the third instars in Si addition treatments in this study may result from the phagostimulating effects of enhanced sugar concentration herein, and/or compensation for a low quality food in accordance with the feeding compensation hypothesis [47,49]. Increased consumption of low quality food may have resulted in lower food conversion efficiency in the rice leaf folder larvae. High sugar concentration impaired development and reduced survival in larvae of *Choristoneura rosaceana* (Harris) [48], which was also evidenced in this study.

Except for the mechanical barrier and food conversion efficiencies mechanisms, recent studies have also shown other Si-modulated plant stress responses to herbivores and pathogens. Si addition induces the activities of plant defensive enzymes such as catalase (CAT), malondialdehyde (MDA), peroxidase (POD), phenylalanine ammonia-lyase (PAL), polyphenol oxidase (PPO) and superoxide dismutase (SOD) [38,50–52] and leads to increased accumulation of defensive compounds such as phenolics, phytoalexins, and momilactones in Si-amended plants [53–55]. In powdery mildew inoculated *Arabidopsis thaliana* plants, Si addition attenuates the decrease in primary metabolism caused by pathogen infection, leading to an overall more efficient defense response [56]. Other reports show that Si addition is associated with increased release of herbivore induced plant volatiles that add to host plant’s defense through attraction of natural enemies of herbivorous insects [57]. More recently, Ye et al. [38] demonstrated a strong interaction between Si and jasmonate (JA) in defense against the rice leaf folder involving priming of JA-mediated defense responses by Si and the promotion of Si accumulation by JA.

The increased food consumption in Si treatments may translate into more damage by the rice leaf folder on Si-treated rice plants. However, the reduced intrinsic rate of increase, finite rate of increase and net reproduction rate of the rice leaf folder population (Table 1) would point to a decreasing size of rice leaf folder populations on Si-treated plants. In China, where half of the paddy fields are Si deficient [58], Si deficiency has become a constraint for sustainable rice production [7,58]. Therefore, with exogenous Si supply to paddy fields, it can be expected that growth and development of rice plants will be improved [7,8] and populations of the rice leaf folder will be reduced. The current results may help to design a suitable integrated pest management program including Si amendment to keep populations of the rice leaf folder below an economically important level.

Collectively, our results clearly demonstrated a Si-mediated resistance to the rice leaf folder in a susceptible rice variety. Si amendment extended larval development and reduced weight gain in third instars, larval survival, pupation rate, and pupal weight. In addition, Si addition reduced the intrinsic rate of increase, finite rate of increase and net reproductive rate of the rice leaf folder. While food consumption was increased, food conversion was reduced in Si amended treatments. Low food quality resulted from Si addition may account for the reduced food conversion efficiencies and the Si-mediated resistance to the rice leaf folder.
